# Migration Governance Through Funding: Theoretical, Normative, and Empirical Perspectives

**DOI:** 10.1080/15562948.2024.2407584

**Published:** 2025-01-03

**Authors:** Evangelia (Lilian) Tsourdi, Federica Zardo

**Affiliations:** a Maastricht University, Maastricht, The Netherlands; b Danube University Krems, Austria

**Keywords:** Funding, externalization, conditionality, migration, governance, financialization, accountability

## Abstract

This special issue explores migration governance through funding while providing unique empirical insights into its design, rationale, use and implications. We embed the rise of funding as a governance tool within the broader debate on the externalization of migration control and the role of conditionality in inter-state relations. We then inductively identify three distinct types of migration governance through funding: earmarking, conditionality, and the whole of government approach. Finally, we explore the impact of governance through funding on migration governance and on diplomacy more broadly, highlighting three dimensions: accountability, transparency, and effectiveness.

## Introduction

Migration has become a particularly contentious issue across the Global North, with governments intensifying their focus on managing migration, often by restricting it. Rising concerns over national security, cultural identity, and economic stability seem to drive this shift, exacerbated by the perception of a significant increase in asylum applications (Dennison & Geddes, 2019; Sahin-Mencutek et al., 2022). In this context, the EU, the USA, and Australia have increasingly co-opted third states in migration management efforts through a process of externalizing core state functions such as border control and asylum processing (Bialasiewicz, 2012; Gammeltoft-Hansen, 2011, Tsourdi & Costello, 2021).

While this phenomenon is not new, it has intensified quantitively and qualitatively over time. Governments have gradually broadened the focus of their migration management efforts from their immediate neighborhoods to regions further along the chain of transit, such as Chad, Niger, or Nauru in Micronesia. Qualitatively, we have witnessed a shift in the deterrence paradigm. Authorities are pursuing the prevention of spontaneous arrivals and the deflection of flows to other destinations with a renewed impetus. A parallel trend of hindering the exit of migrants from third states is rising. Scholars have conceptualized these practices as “contactless control” (Giuffré & Moreno-Lax, 2019; Moreno-Lax & & Others, 2020). This is made possible by outsourcing preemptive migration control beyond the physical borders of destination countries. Such techniques have turned the border into an individualized “shifting border” (Schahar, 2020).

Externalization depends heavily on conditionality, a strategy where third country actors, governmental, non-governmental, and private, are rewarded for effectively pursuing migration management objectives. These rewards can come in the form of funding, visa facilitation, or enhanced opportunities for cooperation in areas beyond migration such as trade, creating an incentive structure that eventually prioritizes migration control. The significant growth in the volume of European, American and Australian funding for migration-related activities beyond their territorial borders over the years is inscribed in this trajectory.

Funding has traditionally been used to share migration responsibilities (Lebon-McGregor & Micinski, 2021; Väyrynen, 2001). Alongside capacity-building and protection, however, countries in the Global North increasingly use funding instrumentally, to pursue migration management objectives through externalization practices (Tsourdi, Zardo & Sayed, 2023; contributions to this special issue). This special issue seeks to capture these dynamics and addresses the following overarching questions:

1. How has funding evolved as a tool in the international governance of migration?
2. How has this evolution impacted state relations, responsibility sharing, and migrants’ rights?
3. How do the above developments reshape the governance of migration?

Answering these questions is necessary because, despite its salience, funding for migration governance has received relatively limited academic attention. Its expansion and its implications are under-theorized, and empirical insights are scarce. Moreover, whenever studied, funds have been mostly treated as a mere corollary of the policy-making process. Instead, funds form the complex link between policy formulation and implementation, involving a combination of political and technocratic arenas, actors, and interests. This gap is relevant because by operationalizing policy objectives, allocating money to specific priorities, sharing implementation responsibilities between actors, and setting the indicators to measure the success of interventions, funds are a crucial non-regulatory policy instrument. They may signal the pace and the direction of governments’ activities and be a proxy for the salience of a particular issue for decision-makers.

Bridging this gap and answering the call voiced by civil society organizations and institutions like the European Parliament to “follow the money” (Open Democracy, 2016), the articles in this special issue provide unique empirical insights into the design, rationale, use and implications of funding to govern migration. Combining political science, legal, and historical perspectives, the contributions assess what the instrumental use of migration funding reveals about the European (EU), American, and Australian approaches to migration and asylum, global responsibility sharing, and the changing nature of migration diplomacy. They also explore the ethical ramifications of funds as a governance tool, analyzing how they may impact migrants’ rights, foster or hinder global responsibility sharing, and affect the rule of law.

The remainder of this article first situates the rise of funding as a governance tool within the broader debate on the externalization of migration control and the role of conditionality. Leveraging insights from the special issue’s contributions, we then propose an evolving typology of governance through funding. Next, we explore the impact of governance through funding on three aspects that emerged from the analysis: accountability, transparency, and effectiveness. A concluding section brings together key arguments and points toward potential directions for future research.

## Beyond externalization and conditionality: the emerging autonomy of funding

External cooperation on migration has deep historical roots, dating to colonial and imperial practices of population management, labor recruitment, and border control (Glynn, 2022; Zolberg 1997). The externalization of migration control observed in the past decades, however, also involves the delegation or outsourcing of migration control to third countries or other external entities. European immigration officers stationed at transit hubs, the European Union’s border agency Frontex coordinating operations outside Europe’s territorial waters, and agreements with third countries to manage or deter migration flows are examples of this strategy in action.

Externalization refers to a strategy encompassing extraterritorial actions aimed at preventing migrants, including asylum seekers, from entering destination countries or regions. It involves a combination of unilateral, bilateral, and multilateral engagements, as well as the incorporation of private actors into migration management practices (Gammeltoft-Hansen 2011; Moreno-Lax, 2017). Examples can be found in major Global North destination areas such as Europe, the USA, and Australia, each adopting unique approaches based on their geopolitical situations and migration challenges but, as this special issue illustrates, also sharing many similarities, in terms of both practices and impacts.

In Europe, the Italian-Libyan Friendship Treaty of 2008 marked a significant shift toward engaging third countries in the control of migration toward Europe (Bialasiewicz, 2012). This agreement, which exchanged financial aid for cooperation in intercepting and returning migrants, exemplifies the broader European strategy of leveraging relationships with countries proximate to migration routes to manage and reduce the flow of migrants. Prior to that experience, Italy had signed ship-rider agreements with Albania in the 1990s to take on board Albanian immigration officers on vessels to interdict Albanian migrants in the Adriatic Sea (Gammeltoft-Hansen 2011).

In the United States, agreements and operations aimed at intercepting migrants before they reach U.S. shores have characterized externalization since the 1980s. This has notably materialized through interdictions in the high seas and collaborations with countries such as Mexico under initiatives such as the Mérida Initiative which integrated migration management measures into counterdrug assistance. Australia’s approach to externalization focused on preventing asylum seekers from arriving by boat through policies like offshore processing centers and bilateral agreements with states such as Nauru and Papua New Guinea (Frelick, Kysel, and Podkul 2016).

Unlike the EU, externalization in the cases of Australia and the USA has also supported offshoring policies. By offshoring, we refer to various policies that could involve either the transfer or the containment of asylum seekers in a third country to undergo admissibility, refugee status determination procedures, or other immigration processing of the offshoring country. Funding plays a key role in securing the third countries’ agreement to implement offshoring processes. Offshoring is financed mainly by the offshoring state rather than the third country government. In addition, the offshoring state often presents further financial incentives to third countries, such as connecting offshoring with accessing funds for development. As Tubakovic and Nethery analyze, Australia has extensively engaged third country governments in such arrangements since 2001 (see Tubakovic and Nethery, this special issue). The Trump administration’s Migrant Protection Protocols, also known as the ‘Remain in Mexico’ policy, is another illustrative example (see Martin-Gil, this special issue).

After Brexit, the UK sought to implement the offshoring paradigm through a judicially contested Memorandum of Understanding, and since December 2023, a treaty with Rwanda (see Matera et al., 2023; House of Lords International Agreements Committee, 2024). In July 2024, the newly elected Labor government announced it would halt these plans (Macaskill & Ravikumar, 2024). While offshoring policies have been debated in the EU for over two decades (e.g. UK Government, 2003; European Council, 2018), they have not materialized. A recent Italy-Albania Protocol seeks to instate such an arrangement between the two countries, but its legality and compatibility with EU law are contested (Carrera et al., 2023; De Leo, 2023).

A variety of interests shape externalization. For destination states, externalization offers a means to manage migration flows, deter irregular migration, and address security and (arguably) humanitarian concerns at a distance. Additionally, governments or states might resort to externalization driven by domestic factors. This includes sidestepping political, normative, and institutional hurdles that constrain policymaking by transferring certain responsibilities to the realm of foreign policy cooperation (Lavenex, 2006). For transit and origin countries, financial aid, infrastructure development, and political support from donor countries can incentivize engagement in externalization policies. In turn, externalization cooperation often provides partner countries with more leverage in international cooperation.

In this framework, conditionality became a pivotal element of international cooperation on migration. Conditionality refers to the strategy whereby destination countries of the Global North link the provision of financial aid, development assistance, and political support to transit and origin countries’ willingness to cooperate in managing and controlling migration flows. This effectively makes cooperation a prerequisite for accessing various forms of assistance, thereby ensuring that transit and origin countries align their policies with the migration control objectives of the destination countries. In his contribution to this special issue, Cassarino illustrates how conditionality may also be instrumentalized to signal unity and “coherence” in the external approach to migration management. The European Commission, for instance, used conditionality to show to the EU member states the added value of its supranational interventions in migration and asylum policies (Cassarino, this special issue).

Funds have long been a key component of states’ toolkits for supporting externalization processes. Incentives for cooperation can include visa facilitation agreements and privileged labor quotas, or the implied possibility of withholding development aid, trade agreements or political cooperation. As with the Italian-Libyan agreements, conditionality may be linked with substantial economic concessions.

The contributions to this special issue highlight three significant trends that demand further attention and research, as they represent a strategic shift toward using economic leverage to influence migration practices abroad. The first is the increase in the amount of funds Global North countries allocated for migration management beyond their borders (European Court of Auditors 2016). For example, by 2023 the European Union’s budget for migration in its development programs surged by a factor of fifty since 2000 (from around 40 million to 2 billion Euros) (see Zardo in this special issue). The second trend is that funding has risen to be the primary method to support externalization practices, outpacing alternative tools such as political dialogues or formal treaties (see, for example Cassarino concerning the EU and Tubakovic and Nethery concerning Australia in this special issue). The third trend is that funds aimed at managing migration are increasingly integrated into programs that do not primarily focus on migration (see for example Zardo concerning the EU, and Martin Gil concerning the USA, in this special issue). This means that the externalization agenda may potentially gain a foothold across a broad spectrum of international cooperation programs and policies, from environmental initiatives to research and agricultural cooperation.

This prioritization of funding as a method to support externalization practices suggests a move toward even more covert, flexible, and possibly unilateral forms of migration governance. Unlike formal treaties or political dialogues, which require negotiation and consensus, and are subject to public scrutiny, funding allows donor states to exert influence more subtly and swiftly. This development mirrors a conceptual shift toward an explicitly transactional approach to the international governance of migration and raises questions about state sovereignty as well as accountability, both in donor and in recipient countries.

Nevertheless, the design and implementation of funding instruments are also subject to internal negotiations and tensions (Capano & Howlett, 2020). The contributions to this special issue highlight the importance of engaging with the rationales and motivations of those actors—whether they are civil servants designing development aid, or international organizations like UNHCR or IOM deploying funds on the ground (see e.g. Ulusoy as well as Sayed in this special issue). Moreover, financial tools may reinforce or undermine each other, feed back into the policymaking process (Zardo, this special issue) and even transform externalization strategies (Gazzotti et al., 2023).

## Governing migration through funding: an evolving typology

Studying funds as governance tools depends on understanding the fundamental nature of these policy instruments (Howlett, 2000; Turnpenny et al., 2015). The latter effort requires categorizing tools as ‘procedural’ or ‘substantive’, or ‘implementation’ versus ‘non-implementation’ oriented. Trauner and Wolff contributed significantly to this categorization. They presented a typology of policy instruments used in the external dimension of the EU migration policy, encompassing agreement- and incentive-based instruments, operational and practical support, and the development of international law and norms. While funding was not a main focus of their work, they categorized funds mainly as the operational component of agreements. Their work highlighted that many of these instruments drew on national or international forerunners in a kind of policy transfer or lesson drawing (Benson & Jordan, 2011; Trauner & Wolff, 2014).

As the contributions in this issue highlight, governments often utilize funds as a non-regulatory instrument to accomplish their policy objectives. Funds provide states with a flexible tool that can be customized to tackle particular challenges. Like all other policy tools, they are complex manifestations of political choices and power dynamics (Zardo, this special issue). The historical precedents of using funds to govern migration imply that the types of funding tools and their design have evolved. In other policy realms such as economy or environment, academic literature has examined the evolution of instrument choices, reflecting on their drivers and implications (Capano & Lippi, 2017; Lascoumes & Le Gales, 2007). Changes may reflect shifts in policy objectives, different interpretations of the same concept and what has been called “the absence of a structured externalization front” (Gazzotti et al., 2023).

In the following analysis, we identify three types of migration governance through funding, categorized by the degree of alignment between the funding and the corresponding policy objectives: earmarking, conditionality, and the whole of government approach. Although conceptually distinct, these types often overlap in time and space. Earmarking represents a traditional mechanism characterized by high specificity. Funds are allocated for specific projects, regions, or programs, with monitoring and evaluation to ensure that resources are used as intended. Conditionality involves linking policy objectives to funding, with financial support contingent upon the recipient meeting specific conditions. Moderate specificity characterizes this approach. The whole of government approach features medium to low specificity, integrating funding into a broader strategy, encompassing various government departments and agencies. Rather than constructing a strict taxonomy, we intend to use this categorization as a heuristic tool to better understand the development of governance through funding and its impact over time and across regions.

### Earmarking

Earmarking broadly refers to tying financial resources to specific objectives, projects, or geographic regions. In the international organizations (IO) scholarship, earmarked funding is juxtaposed with mandatory funding schemes that require member states to make financial contributions to the IO (Graham, 2015). The UN and several UN specialized agencies vary mandatory contributions based on each state’s capacity to pay. Mandatory funding is then dispersed across programs based on collective decisionmaking, for example through decisions of the UN General Assembly. Voluntary funding is additional to the mandatory contributions and can take two forms: unrestricted funding where donors do not earmark contributions for a specific use, and restricted funding which allows donors, which depending on the IO’s internal rules could also include non-state donors, to earmark funds (Graham, 2015).

Earmarking has distinct steering potential as donors seek to pursue specific objectives. Nevertheless, funding and influence are bidirectional, as for example IOs can influence funding patterns as well (Michaelowa, 2017). Scholars have argued that the rise of earmarked funding in international governance has the potential to undermine multilateralism (Bayram & Graham, 2017; Goetz & Patz, 2017; Graham, 2017a; Seitz & Martens, 2017). At the same time, scholarship has linked funding to IO empowerment, as the more financial resources available to an IO, the greater its power (Heldt & Schmidtke, 2017). Longitudinal data indicates that it is states with favorable views of the UN that have sought to expand its activities by introducing flexible funding rules and more control by donors (Graham, 2017b).

Earmarked funding plays a significant role in migration governance, with voluntary earmarked contributions annually representing the main source of revenue for major international agencies such as UNHCR and the IOM (Lebon-McGregor & Micinski, 2021). Earmarking can concern a specific type of activity or a specific geographic focus. For example, the USA earmarked funding to UNHCR in the 1990s to address the situation of Central American migrants transiting through Mexico (Martin Gil, this special issue). The IOM largely receives funding earmarked for projects, which has led commentators to argue that donors shape its global operations (Bradley et al., 2023; Pécoud, 2018). Longitudinal quantitative research largely supports these arguments (Lebon-McGregor & Micinski, 2021; Patz & Thorvaldsdottir, 2020), and has further illustrated that despite its reliance on earmarked funding, UNHCR is able to allocate funds broadly in line with its mandate, with donor interests having, at most, inconsistent influence on their distribution (Thorvaldsdottir et al., 2022).

Earmarking is employed in all regions this special issue studies. For example, the USA earmarked funding for migration management projects within counterdrug funding directed to Mexico (Martin Gil, this special issue). Australia earmarked funding to the governments of Nauru and Papua New Guinea for the construction and running of immigration detention facilities (Tubakovic and Nethery, this special issue). Within EU funds, earmarking is prevalent, in the sense that funding lines pursue specific objectives; for example, the regulation establishing the EU’s Asylum, Migration and Integration Fund contains an exhaustive list of broadly phrased pre-defined objectives that the funding should pursue (Article 3, AMIF Regulation, 2021). However, earmarking is also used as a steering technique within the Asylum, Migration and Integration Fund to ensure balance among the different objectives, or to prioritize some objectives (e.g. Article 11, AMIF Regulation, 2021). Migration funding is also earmarked to specific national contexts, such as the Facility for Refugees in Turkey (Ulusoy, this special issue), or development funding specifically targeting Afghanistan prior to its 2021 takeover by the Taliban (Sayed, this special issue), or the previous EU Trust Funds, for example the Trust Fund for Africa.

The practice of including migration management objectives within development funds has also evolved. Previously, the ad hoc EU Trust Funds did not allocate a specific percentage for these objectives. In contrast, the current EU fund for development cooperation, the Neighborhood Development and Cooperation Instrument (NDICI), mandates that 10% of its financial resources be dedicated to addressing migration and forced displacement. The NDICI approach suggests a shift toward earmarking, which ties funding to measurable progress on migration cooperation. Despite this, the EU’s method for identifying the “migration relevance” of EU-funded interventions has not significantly improved the traceability and monitoring of these interventions.

In 2021, the European Commission introduced a migration marker to determine whether funding counts toward the NDICÍs 10% spending target. Zardo’s analysis in this special issue shows that even project documents without the migration marker eventually included migration-relevant actions (Zardo, this special ssue, see also Zardo and Tservenis, forthcoming, 2024). The conservative approach to classifying activities as related to migration (Woollard et al., 2022, p. 51) is due to a lack of coordination within the Commission, uncertainty about whether migration is a primary objective or merely part of broader development interventions, and the administrative need to balance and monitor earmarking activities to ensure alignment with overarching goals (Zardo, this special issue).

### Conditionality

Conditionality in foreign aid involves linking financial support to specific policy objectives, and to the recipient meeting certain conditions. This approach can take the form of either positive incentives or negative sanctions. Positive conditionality rewards countries for making progress on agreed goals, such as improving migration management, while negative conditionality withdraws or withholds aid if these conditions are not met. This strategy has become a central component of the relationships between countries of destination, origin, and transit of migrants and refugees (Cassarino, 2005, 2014). Governments of destination countries have increasingly leveraged their financial support and development assistance to influence migration policies and border control practices in origin and transit countries.

While migration control policies and practices are gradually converging across Western countries (Martin Gil, this special issue; Meyers, 2002), and reliance on conditionality to steer cooperation on migration is a common pattern, its implementation varies across regions. During the 1980s, US foreign aid to Central America was linked to countering Soviet influence and narcotics control, with a secondary aim of deterring migration. As Martin Gil shows, the Reagan administration, for instance, provided substantial military aid to El Salvador under the condition that the Salvadoran government would cooperate in preventing the northward flow of refugees (Martin Gil, this special issue).

This approach intensified over the years, with aid increasingly tied to strict migration enforcement measures. In the 1990s, the USA pressured Mexico to act as a buffer state, deporting undocumented migrants in transit to the USA. By the 2000s, migration control in Mexico and Central America became closely linked to counternarcotics assistance through initiatives like the Mérida Initiative, which included US funding for biometric and immigration control equipment (Martin Gil, this special issue). The Trump applied negative conditionality in 2019 when it suspended foreign aid to El Salvador, Guatemala, and Honduras until they signed Asylum Cooperative Agreements (ACAs), effectively shifting asylum responsibilities to these countries (Martin Gil, this special issue).

The EU has largely avoided negative conditionality in its external relations, instead adopting a “more for more” approach. Negative conditionality may occur, though it is less publicly discussed and often used in negotiations without being explicitly enforced. The EU revised its Mobility Partnership with Cabo Verde to include a clause that could suspend the Visa Facilitation Agreement (VFA) for insufficient cooperation in readmissions. There have been instances of negative conditionality concerning Afghanistan, Ghana, Nigeria, and Pakistan, where financial aid was threatened or withdrawn to ensure compliance with migration management standards (Lebon-McGregor et al., this special issue).

Conditionality initially aimed at promoting democratization and stability, and subsequently at progress in migration management objectives. In his analysis, Cassarino notes that the European Neighborhood Policy (ENP), launched in 2004, was a pivotal moment in the shift toward migration related conditionality (Cassarino, this special issue). The ENP aimed to foster stability, security, and prosperity in neighboring regions, including key transit areas like North Africa. However, over time, the policy increasingly linked its development component to migration management, requiring recipient countries to enhance their border controls and cooperate in readmission agreements. This marked the beginning of a more explicit and structured approach to conditionality, where financial assistance was directly tied to the recipient country’s willingness and ability to manage migration flows in line with EU interests (Cassarino, this special issue).

The 2016 European Agenda for Migration further consolidated the EU’s use of conditionality, introducing the Migration Partnership Framework (MPF). As Collet and Ahad outline (2017:4), this framework is “the second-generation partnership approach,” which emphasized increased conditionality and compensation (Strik, 2017). The current EU fund for development cooperation, the NDICI, exemplifies this trend. It includes a ‘flexible incitative approach’ that unlocks an additional 10% of the budget for countries showing progress in migration cooperation.

Conditional funding can be politically expedient, allowing donor governments to address domestic political concerns by making aid contingent on recipient countries’ good behavior. This helps mitigate criticisms of wasteful or unaccountable spending by providing a sense of control over how aid is used. The design of funding programs targeting migration often aims to communicate an “appearance of control” to constituencies (Zardo, this special issue). Governments believe they can ensure that aid is not misappropriated or ineffective by tying it to specific conditions and monitoring compliance. Moreover, conditionality is a well-established practice in international relations and development aid. Its widespread use by major international organizations, such as the International Monetary Fund (IMF) and the World Bank, lends it legitimacy and encourages governments to adopt similar approaches in a politically salient realm such as migration.

### The whole of government approach

The blurring of boundaries between development cooperation, humanitarian aid, and migration control is a consistent thread across regions and countries. This characteristic underpins the Whole of Government Approach (WGA), where multiple government departments and agencies collaborate to address complex policy issues. We differentiate WGA from conditionality in terms of the breadth of the issues linked, and of the depth of the integrated multi-stakeholder collaboration. Notably, recent cooperation frameworks of the EU and its member states with Egypt (European Commission, 2024) and Lebanon (European Commission, 2023) link disparate policy areas including macroeconomic stability, digital transition, environmental transition, trade, security, and migration management, while involving various sources of funding and multiple public and private stakeholders.

The USA’s investments in Central America under the “Root Causes of Migration Strategy” are another example of the WGA. These investments aim at improving living conditions in migrant-origin regions, including funding for anticorruption, law enforcement professionalization, and justice sector programs. These efforts reflect a broader strategic reorientation of migration policies, leveraging financial aid to effect systemic changes in asylum systems of developing countries, potentially transforming them into destination countries. Another example is the Biden administration’s “Collaborative Migration Management Strategy” which enhances humanitarian aid to Guatemala, El Salvador, and Honduras, while providing financial support to UNHCR for developing and expanding asylum systems in Guatemala and Mexico.

Within the WGA, the increasing financialization of migration governance involves using financial instruments such as loans, guarantees, and equity participation to leverage public and private investments. Initially introduced in sectors like SME support, research, innovation, and environmental sustainability to address market failures or suboptimal investment conditions, these instruments have also found application in migration governance (Bakker, 2010; Davitti, 2023; Kunz et al., 2022). Financialization was also present under some earlier schemes involving conditionality arrangements that linked development aid to migration management, for example under the EU Trust Funds (Zardo 2022). However, it can be conceptualized as an integral part of the WGA as it complements budgetary resources and provides strategic support to build capacities for pooling resources and developing coherent actions (European Commission, 2022). This economic-centric view risks overshadowing the humanitarian and social dimensions of migration, as it might prioritize financially lucrative projects over those most needed from a humanitarian perspective (Davitti, 2023).

The interaction between complementary pathways to legal migration and mechanisms of refugee finance illustrates how financialization might increase the precarity and temporariness of protection. This interaction can challenge the definition of refugee protection in a context where private investors become key enablers and co-providers of protection (Vankova and Davitti, this special issue). Despite these challenges, governmental actors in the EU and the USA have accelerated the process of financialization to pursue migration management strategies embedded within broader cooperation frameworks.

## Impact of governance through funding

Governance through funding has profound impacts on migration governance and on diplomacy more broadly. We highlight three dimensions of impact: accountability, transparency, and effectiveness.

### Accountability

Accountability emerges as a major challenge as states can mobilize governance through funding precisely to eschew accountability. Tubakovic and Nethery argue that Australia’s offshoring activities can be conceptualized as an instance of ‘attenuated governance’ (Tubakovic and Nethery, this special issue), a governance mode that also evades accountability for fundamental rights violations. Authors in this special issue also explore how governance through funding has been linked with the rise of fundamental rights violations in for example Libya (De Leo, this special issue) and Turkey (Ulusoy, this special issue) through EU funded cooperation frameworks, as well as in Mexico (Ardalan, this special issue) through US funded cooperation frameworks.

Ensuring judicial accountability for fundamental rights violations linked to governance through funding is proving difficult. Contributions to this special issue illustrate the difficulties of judicially challenging the operationalization of offshoring arrangements and the application of safe third country concepts underpinned by funding in Australia (Tubakovic and Nethery, this special issue), the USA (Ardalan, this special issue), and the EU (Ulusoy and De Leo, this special issue). An additional challenge is that current interpretations of international law do not cover the factual situations of providing operational and financial support to third countries that are responsible for the fundamental rights violations (De Leo, this special issue, Tsourdi & De Bruycker, 2022). Scholars have proposed innovative interpretations to expand the scope of jurisdiction (Moreno-Lax, 2020), but a relevant decision from the European Court of Human Rights is still lacking.

Administrative and financial accountability avenues are being explored as complementary alternatives. The submission of an informal complaint concerning the financing of Libya to the European Court of Auditors illustrates an attempt to redress insufficient accountability for violations of fundamental rights that occur during the implementation of EU funded activities (De Leo, this special issue). The European Ombudsman, an independent institution that investigates instances of maladministration by EU institutions, offers another potential avenue for accountability (De Leo, this special issue).

### Transparency

Lack of transparency is another impact of governing migration through funding. Transparency in the allocation and execution of funding programs is not just a matter of bureaucratic oversight. It affects how international migration policies are conceptualized, implemented, and experienced on the ground. Previous scholarship has analyzed how local actors are often best positioned to respond to needs on the ground, and their marginalization can lead to less effective interventions, disconnected from the realities of those they aim to serve (Sobhan, 2002).

In this special issue, contributions illustrate how the centralized approach to funding has marginalized local NGOs and civil society, diminishing the effectiveness and sustainability of programs. For example, the European Union’s Facility for Refugees in Turkey (FRiT) significantly sidelined local NGOs, despite their being best positioned to respond to needs on the ground (Ulusoy, this special issue). In Afghanistan, EU interests have driven the overarching design of EU-funded programs, but there has been some strategic steering of national or regional projects to align with EU migration control objectives. This raises questions about the autonomy of local policies and the extent to which external funding influences national priorities (Sayed, this special issue).

Lack of transparency is also linked with limited traceability of funds. In the USA, as Ardalan explains, lack of transparency in soft cooperation frameworks has led to the Department of Homeland Security (DHS) misallocating State Department funds to conduct operations in third countries that seek to halt the onward movement of Honduran migrants from Guatemala (see Ardalan, this special issue and the sources she cites). In the EU, the patchwork of funding lines employed makes it difficult to trace the overall amount of funding received by third countries for operationalizing the EU’s external migration policy (Woollard et al., 2022).

The consolidation of several development-related funding lines into the current EU fund for development cooperation, the NDICI, is a step toward increased transparency. However, challenges remain, as the patchwork of funds targeting the external dimension of migration persists. For instance, internal funding from Ministries of the Interior can also target external actions on migration, yet there is no consolidated oversight of migration related spending across EU funding programs (Zardo, this special issue). The rise of multi-stakeholder funding schemes, such as the envisaged Talent Partnerships with third countries, which are expected to combine public and private funding, further compounds the transparency challenge. Hence, we have proposed elsewhere how traceability could be increased in this framework, for example, through the creation of a centralized registration system for Talent Partnerships (Tsourdi et al., 2023)

### Effectiveness

Finally, contributions in this special issue speak to the effectiveness of governance through funding. By effectiveness we refer to funding’s steering potential, the extent to which the activities undertaken achieve the objectives pursued. Empirical findings in this special issue present evidence of the effectiveness of governance through funding. For example, EU funding has played an important role in steering national, sub-national, or sub-regional programs and projects in Afghanistan to pursue EU migration-related objectives (Sayed, this special issue). In addition, EU funding has improved the living conditions, access to services and social cohesion of refugees and asylum seekers (Ulusoy, this special issue). However, in line with previous research, overreliance on governance through funding without robust mechanisms for enforcement and accountability has also led to superficial compliance, and undermined long term objectives such as development (Blauberger & van Hüllen, 2021; Sayed, this special issue).

In line with decentering approaches to international relations (Onar & Nicolaïdis, 2013; Wolff et al., 2022), contributors in this special issue move beyond exclusively Eurocentric frames to assess how partner countries exercise their agency when involved in governance through funding frameworks, and the impact of such agency. Cassarino analyzes how patterns of interdependence between EU and non-EU countries produce unintended consequences for the EU’s external relations. Notably, third countries have leveraged non-cooperation on migration through ‘reverse conditionalities’, aiming to defend their interests and preferences (Cassarino, this special issue). Ulusoy reflects on the impact of governance through funding in Turkey which has also led to the commodification of migrants and refugees and transformed cooperation on migration into pervasive and routinized transactional relationships (Ulusoy, this special issue). Martín Gil traces how Mexico leveraged the Trump administration’s urge for migrant containment to secure increased development aid (Martín Gil, this special issue).

The analysis in this special issue resonates with scholarship on refugee rentier states, on migration bureaucratic politics, and on weaponized migration. Tsourapas has conceptualized how states in the Global South employ refugee ‘rent seeking’ strategies (Tsourapas, 2019), i.e. leverage their position to attract funding in exchange for maintaining refugees within their territories. Further scholarship has applied this framework to explore how refugee rent seeking behavior is diffused across regions (Freier et al., 2021), as well as how subnational actors (Dhingra, 2024) and sub-state groups (Yassen & McGee, 2024) seek to extract refugee rent. In addition, scholarship on West African states has conceptualized migration policymaking as an ‘intermestic’ policy issue, reflecting the dynamic interplay of international and domestic interests (Adam et al., 2020), and has analyzed how migration cooperation at the same time empowers and renders dependent national bureaucracies in partner countries (Roos et al., 2024). Finally, security study scholars have examined how pervasive and routinized transactional relationships have led to engineering cross-border population movements, going beyond pure realpolitik (Adamson & Greenhill, 2023; Greenhill, 2008).

## Conclusion

Governing migration through funding is on the rise across various regions. Initially deployed as instruments of solidarity, funds have evolved into significant tools for migration governance and play a crucial role in supporting externalization. This shift in scope has broadened the spectrum of actors, arenas, and interests engaged in the design and execution of funding mechanisms, thereby adding layers of complexity for researchers striving to decipher what public policy scholars call the instrumentation process (Lascoumes & Le Gales, 2007).

We have inductively identified three distinct types of migration governance through funding that we categorized according to the degree of alignment between the funding and the corresponding policy objectives: earmarking, conditionality, and the whole of government approach. While they often overlap in time and space, such categorization helped us to identify how each mechanism operates, its patterns, and its impacts. Earmarking involves tying financial resources to specific objectives, projects, or geographic regions, as seen in the EU’s current EU fund for development cooperation, the NDICI, which mandates that 10% of its financial resources address migration and forced displacement. Conditionality, as in the imposition of incentives or sanctions relating migration management to financing, trade, or diplomatic support, has become a centerpiece of governance through funding. The whole of government approach streamlines migration management objectives in broader inter-state relations, linking migration management goals, funding, and actors to disparate policy areas such as macroeconomic stability, digital transition or environmental transition, as observed in the recent cooperation frameworks of the EU with Egypt and Lebanon.

Contributions in this special issue trace and analyze the distinct impact of governance through funding on accountability, transparency, and policy effectiveness. Judicial accountability for fundamental rights violations linked with migration governance through funding remains somewhat elusive due to jurisdiction, standing, and practical barriers. Nonetheless, strategic litigation efforts are evolving. A more recent trend is having recourse to administrative and financial accountability mechanisms as complementary and more flexible avenues to judicial recourse. Transparency issues are pervasive in most contexts, partly due to policy design and partly because of the inherent complexity of the actors and resources involved.

Finally, empirical insights attest to the steering potential of funding and the enhancement of policy effectiveness through funding. Nonetheless, overreliance on migration governance through funding has also led to superficial compliance, as well as undermined long-term objectives such as development. In addition, effectiveness lies in the eye of the beholder. Governance through funding generates intricate interdependencies that empower partner countries, which also exercise their agency to defend their own interests and preferences when involved in such cooperation frameworks. A characteristic example is when partner countries leverage non-cooperation on migration through ‘reverse conditionalities’, reversing the flow of influence.

Further research could address the selection, combination, and deployment of funds and funding schemes in migration governance. Investigating how funding, for instance, interacts with other instruments in the migration realm and how different programs affect each other is crucial. Moreover, more attention could be paid to identifying how third countries and, more broadly, recipients such as bureaucrats and civil society organizations react to different types of tools. Understanding the motivations, incentives, and potential resistances not only to policies (Roos et al., 2024) but to programs and projects on the ground would also advance the research agenda on policy implementation. The normative, theoretical, and empirical insights included in this special issue pave the way for such future research.

## References

[CIT0001] Adam, I., Trauner, F., Jegen, L., & Roos, C. (2020). West African interests in (EU) migration policy. Balancing domestic priorities with external incentives. *Journal of Ethnic and Migration Studies*, *46*(15), 3101–3118. 10.1080/1369183X.2020.1750354

[CIT0002] Adamson, F. B., & Greenhill, K. M. (2023). Deal-making, diplomacy and transactional forced migration. *International Affairs*, *99*(2), 707–725. 10.1093/ia/iiad017

[CIT0003] AMIF Regulation. (2021). Regulation (EU) 2021/1147 of the European Parliament and of the Council of 7 July 2021 establishing the Asylum, Migration and Integration Fund. OJ L 251/1.

[CIT0004] Bakker, M. (2010). From ‘The Whole Enchilada’ to financialization: Shifting discourses of migration management in North America. In M. Geiger & A. Pécoud (Eds.), *The politics of international migration management* (pp. 271–294). Palgrave Macmillan UK. 10.1057/9780230294882_13

[CIT0005] Bayram, A. B., & Graham, E. R. (2017). Financing the United Nations: Explaining variation in how donors provide funding to the UN. *The Review of International Organizations*, *12*(3), 421–459. 10.1007/s11558-016-9261-0

[CIT0006] Benson, D., & Jordan, A. (2011). What have we learned from policy transfer research? Dolowitz and marsh revisited. *Political Studies Review*, *9*(3), 366–378. 10.1111/j.1478-9302.2011.00240.x

[CIT0007] Bialasiewicz, L. (2012). Off-shoring and out-sourcing the borders of EUrope: Libya and EU border work in the mediterranean. *Geopolitics*, *17*(4), 843–866. 10.1080/14650045.2012.660579

[CIT0008] Blauberger, M., & van Hüllen, V. (2021). Conditionality of EU funds: An instrument to enforce EU fundamental values? *Journal of European Integration*, *43*(1), 1–16. 10.1080/07036337.2019.1708337

[CIT0009] Bradley, M., Costello, C., & Sherwood, A. (Eds.). (2023). *IOM Unbound?: Obligations and Accountability of the International Organization for Migration in an Era of Expansion*. Cambridge University Press.

[CIT0010] Capano, G., & Howlett, M. (2020). The Knowns and unknowns of policy instrument analysis: Policy tools and the current research agenda on policy mixes. *SAGE Open*, *10*(1), 215824401990056. 10.1177/2158244019900568

[CIT0011] Capano, G., & Lippi, A. (2017). How policy instruments are chosen: Patterns of decision makers’ choices. *Policy Sciences*, *50*(2), 269–293. 10.1007/s11077-016-9267-8

[CIT0012] Carrera, S., Campesi, G., & Colombi, D. (2023). *The 2023 Italy-Albania protocol on extraterritorial migration management*. https://www.ceps.eu/ceps-publications/the-2023-italy-albania-protocol-on-extraterritorial-migration-management/

[CIT0013] Cassarino, J.-P. (2005). Migration and border management in the Euro-Mediterranean Area: Heading towards new forms of interconnectedness. http://cadmus.eui.eu/handle/1814/6283

[CIT0014] Cassarino, J.-P. (2014). Channelled policy transfers: EU-Tunisia interactions on migration matters. *European Journal of Migration and Law*, *16*(1), 97–123. 10.1163/15718166-00002046

[CIT0015] Davitti, D. (2023). Refugee finance: External(ized) protection and investments for refugees. *Quaderni Della Facoltà Di Giurisprudenza/Series of the Faculty of Law, University of Trento*, *65*, 83–100.

[CIT0016] De Leo, A. (2023). On the incompatibility of the Italy-Albania protocol with EU asylum law. SIDI *Blog*. http://www.sidiblog.org/2023/11/15/on-the-incompatibility-of-the-italy-albania-protocol-with-eu-asylum-law/

[CIT0017] Dennison, J., & Geddes, A. (2019). A rising tide? The salience of immigration and the rise of anti-immigration political parties in Western Europe. *The Political Quarterly*, *90*(1), 107–116. 10.1111/1467-923X.12620

[CIT0018] Dhingra, R. (2024). A tale of two municipalities: The local politics of international assistance during refugee crises. *POMEPS Studies*, *50,*106-114.

[CIT0019] European Commission. (2022). *Attracting skills and talent to the EU*. COM(2022)657. https://eur-lex.europa.eu/legal-content/EN/TXT/?uri=COM:2022:657:FIN

[CIT0020] European Commission. (2022). Fi-compass news—Spring 2022—Financial instruments addressing EU migration challenges. https://www.fi-compass.eu/f/fi-compass-news-spring-2022/financial-instruments-addressing-eu-migration-challenges/

[CIT0021] European Commission. (2023). Memorandum of understanding on a strategic and global partnership between the European Union and Tunisia. https://ec.europa.eu/commission/presscorner/detail/en/ip_23_3887

[CIT0022] European Commission. (2024). Joint declaration on the strategic and comprehensive partnership between the Arab Republic of Egypt and the European Union. https://neighbourhood-enlargement.ec.europa.eu/news/joint-declaration-strategic-and-comprehensive-partnership-between-arab-republic-egypt-and-european-2024-03-17_en

[CIT0023] European Court of Auditors. (2016). *EU external migration spending in southern mediterranean and eastern neighbourhood countries until 2014*. Special Report No 09, 2016. Publications Office of the European Union. https://data.europa.eu/doi/10.2865/18042

[CIT0024a] Freier, L. F., Micinski, N. R., & Tsourapas, G. (2021). Refugee commodification: The diffusion of refugee rent-seeking in the Global South. *Third World Quarterly*, *42*(11), 2747–2766. 10.1080/01436597.2021.1956891

[CIT0024] Frelick, B., Kysel, I. M., & Podkul, J. (2016). The Impact of Externalization of Migration Controls on the Rights of Asylum Seekers and Other Migrants. Journal on Migration and Human Security, 4(4), 190–220. 10.1177/233150241600400402

[CIT0025] Gammeltoft-Hansen, T. (2011). *Access to Asylum: International refugee law and the globalisation of migration control*. Cambridge University Press.

[CIT0026] Gammeltoft-Hansen, T. (2011). The externalisation of european migration control and the reach of international refugee law. *SSRN Electronic Journal (pp. 1-22)*, (SSRN Scholarly Paper 3905769). 10.2139/ssrn.3905769

[CIT0027] Gazzotti, L., Jiménez Álvarez, M. G., & Espiñeira, K. (2023). A “European” externalization strategy? a transnational perspective on aid, border regimes, and the EU trust fund for Africa in Morocco. In C. Finotelli & I. Ponzo (Eds.), *Migration control logics and strategies in Europe: A North-South comparison* (pp. 69–89). Springer International Publishing. 10.1007/978-3-031-26002-5_4

[CIT0028] Giuffré, M., & Moreno-Lax, V. (2019). The rise of consensual containment: From contactless control to contactless responsibility for migratory flows. In S. Juss (Ed.), *Research handbook on international refugee law* (pp. 82–108). Edward Elgar Publishing.

[CIT0029] Glynn, I. (2022). Protecting Australia’s borders since the 1850s: At the cutting edge of border control but on the edge of international acceptability. In *Borders and Mobility Control in and between Empires and Nation-States* (pp. 291–320). Brill. 10.1163/9789004520844_013

[CIT0030] Goetz, K. H., & Patz, R. (2017). Resourcing international organizations: Resource diversification, organizational differentiation, and administrative governance. *Global Policy*, *8*(S5), 5–14. 10.1111/1758-5899.12468

[CIT0031] Graham, E. R. (2015). Money and multilateralism: How funding rules constitute IO governance. *International Theory*, *7*(1), 162–194. 10.1017/S1752971914000414

[CIT0032] Graham, E. R. (2017a). Follow the money: How trends in financing are changing governance at international organizations. *Global Policy*, *8*(S5), 15–25. 10.1111/1758-5899.12450

[CIT0033] Graham, E. R. (2017b). The institutional design of funding rules at international organizations: Explaining the transformation in financing the United Nations. *European Journal of International Relations*, *23*(2), 365–390. 10.1177/1354066116648755

[CIT0034] Greenhill, K. M. (2008). Strategic engineered migration as a Weapon of War. *Civil Wars*, *10*(1), 6–21. 10.1080/13698240701835425

[CIT0035] Heldt, E., & Schmidtke, H. (2017). Measuring the empowerment of international organizations: The evolution of financial and staff capabilities. *Global Policy*, *8*(Suppl 5), 51–61. 10.1111/1758-5899.12449 29213334 PMC5703441

[CIT0036] House of Lords International Agreements Committee. (2014). *Scrutiny of international agreements: UK-Rwanda agreement on an asylum partnership*. https://committees.parliament.uk/publications/42927/documents/213461/default/

[CIT0037] Howlett, M. (2000). Managing the “Hollow State”: Procedural policy instruments and modern governance. *Canadian Public Administration*, *43*(4), 412–431. 10.1111/j.1754-7121.2000.tb01152.x

[CIT0038] Kunz, R., Maisenbacher, J., & Paudel, L. N. (2022). Remittances, development and financialization beyond the global north. *Environment & Planning A*, *54*(4), 693–701. 10.1177/0308518X221088867 35528225 PMC9069657

[CIT0039] Lascoumes, P., & Le Gales, P. (2007). Introduction: Understanding public policy through its instruments—from the nature of instruments to the sociology of public policy instrumentation. *Governance*, *20*(1), 1–21. 10.1111/j.1468-0491.2007.00342.x

[CIT0040] Lavenex, S. (2006). Shifting up and out: The foreign policy of European immigration control. *West European Politics*, *29*(2), 329–350. 10.1080/01402380500512684

[CIT0041] Lebon-McGregor, E., & Micinski, N. (2021). The changing landscape of multilateral financing and global migration governance. In T. de Lange, W. Maas & A. Schrauwen (Eds.), *Money matters in migration: policy, participation, and citizenship* (pp. 19–27). Cambridge University Press.

[CIT0042] Macaskill, A., & Ravikumar, S. (2024). New UK leader Starmer Declares Rwanda deportation plan ‘Dead and Buried’. *Reuters.* https://www.reuters.com/world/uk/new-uk-pm-starmer-confirms-end-rwanda-asylum-deportation-scheme-2024-07-06/

[CIT0043] Matera, M., Tubakovic, T., & Murray, P. (2023). Is Australia a model for the UK? A critical assessment of parallels of cruelty in refugee externalization policies. *Journal of Refugee Studies*, *36*(2), 271–293. 10.1093/jrs/fead016

[CIT0044] Meyers, E. (2002). The causes of convergence in Western immigration control. *Review of International Studies*, *28*(1), 123–141. 10.1017/S0260210502001237

[CIT0045] Michaelowa, K. (2017). Resourcing international organisations: So what? *Global Policy*, *8*(S5), 113–123. 10.1111/1758-5899.12471

[CIT0046] Moreno-Lax, V. (2017). *Accessing Asylum in Europe: Extraterritorial border controls and refugee rights under EU law*. Oxford University Press.

[CIT0047] Moreno-Lax, V. (2020). The architecture of functional jurisdiction: Unpacking contactless control—on public powers, SS and Others v. Italy, and the ‘Operational Model. *German Law Journal*, *21*(3), 385–416. 10.1017/glj.2020.25

[CIT0048] Onar, N. F., & Nicolaïdis, K. (2013). The Decentring agenda: Europe as a post-colonial power. *Cooperation and Conflict*, *48*(2), 283–303. http://www.jstor.org/stable/45084725 10.1177/0010836713485384

[CIT0049] Open Democracy. (2016). Migration – follow the money. openDemocracy. https://www.opendemocracy.net/en/migration-follow-money/

[CIT0050] Patz, R., & Thorvaldsdottir, S. (2020). Drivers of expenditure allocation in the IOM: Refugees, donors, and international bureaucracy. In Pecoud, A., and Geiger, M. (Eds.), *The international organization for migration: The new ‘UN Migration Agency’ in critical perspective* (pp. 75–98). Palgrave Macmillan. https://link.springer.com/chapter/10.1007/978-3-030-32976-1_4

[CIT0051] Pécoud, A. (2018). What do we know about the international organization for migration? *Journal of Ethnic and Migration Studies*, *44*(10), 1621–1638. 10.1080/1369183X.2017.1354028

[CIT0052] Roos, C., Trauner, F., & Adam, I. (2024). Bureaucratic migration politics in West Africa: Opportunities and dependencies created by EU funding. *International Migration Review*, *58*(1), 296–318. 10.1177/01979183221142775

[CIT0053] Sahin-Mencutek, Z., Barthoma, S., Gökalp-Aras, N. E., & Triandafyllidou, A. (2022). A crisis mode in migration governance: Comparative and analytical insights. *Comparative Migration Studies*, *10*(1), 12. 10.1186/s40878-022-00284-2 35371921 PMC8934758

[CIT0054] Schahar, A. (2020). *The shifting border: Legal cartographies of migration and mobility: Ayelet Shachar in dialogue*. Manchester University Press.

[CIT0055] Seitz, K., & Martens, J. (2017). Philanthrolateralism: Private funding and corporate influence in the United Nations. *Global Policy*, *8*(S5), 46–50. 10.1111/1758-5899.12448

[CIT0056] Sobhan, R. (2002). Aid effectiveness and policy ownership. *Development and Change*, *33*(3), 539–548. 10.1111/1467-7660.00267

[CIT0057] Strik, T. (2017). Two decades eu migration law for third country nationals: Are there any integration challenges left?. In *Migration on the move* (pp. 76–92). Brill Nijhoff. 10.1163/9789004330467_006

[CIT0058] Thorvaldsdottir, S., Patz, R., & Goetz, K. H. (2022). Mandate or donors? Explaining the UNHCR’s country-level expenditures from 1967 to 2016. *Political Studies*, *70*(2), 443–464. 10.1177/0032321720974330

[CIT0059] Trauner, F., & Wolff, S. (2014). The negotiation and contestation of EU migration policy instruments: A research framework. *European Journal of Migration and Law*, *16*(1), 1–18. 10.1163/15718166-00002046

[CIT0060] Tsourapas, G. (2019). The Syrian refugee crisis the Syrian refugee crisis and foreign policy decision-making in Jordan, Lebanon, and Turkey. *Journal of Global Security Studies*, *4*(4), 464–481. 10.1093/jogss/ogz016

[CIT0061] Tsourdi, E., & Costello, C. (2021). The evolution of EU law on refugees and asylum. In P. Craig, and G. de Búrca (Eds.), *The evolution of EU law* (3rd edn., pp. 793–823). Oxford University Press. 10.1093/oso/9780192846556.003.0025

[CIT0062] Tsourdi, E., & De Bruycker, P. (2022). The evolving EU asylum and migration law. In E. Tsourdi & P. De Bruycker (Eds.), *Research handbook on EU migration and asylum law* (pp. 1–56). Edward Elgar Publishing.

[CIT0063] Tsourdi, E., Ott, A., & Vankova, Z. (2022). The EU’s shifting borders reconsidered: Externalization, constitutionalization, and administrative integration. *European Papers-A Journal on Law and Integration*, *7*(1), 87–108. 10.15166/2499-8249/549

[CIT0064] Tsourdi, E., Zardo, F., & Sayed, N. (2023). *Funding the EU’s External Migration Policy: ‘Same Old’ or Potential for Sustainable Collaboration?.* European Policy Centre. https://www.epc.eu/en/Publications/Funding-the-EUs-external-migration-policy-Same-old-or-potential-fo∼4fb44c

[CIT0065] Turnpenny, J. R., Jordan, A. J., Benson, D., & Rayner, T. (2015). The Tools of Policy Formulation: An Introduction. In *The tools of policy formulation* (pp. 3–30). Edward Elgar Publishing. https://www.elgaronline.com/edcollchap-oa/edcoll/9781783477036/9781783477036.00011.xml

[CIT0066] UK Government. (2003). *New international approaches to asylum processing and protection*. http://www.statewatch.org/news/2003/apr/blair-simitis-asile.pdf

[CIT0067] Väyrynen, R. (2001). Funding dilemmas in refugee assistance: Political interests and institutional reforms in UNHCR. *International Migration Review*, *35*(1), 143–167. 10.1111/j.1747-7379.2001.tb00009.x

[CIT0068] Wolff, S., Gazsi, D., Huber, D., & Fisher-Onar, N. (2022). How to reflexively decentre EU foreign policy: Dissonance and contrapuntal reconstruction in migration, religious and neighbourhood governance. *JCMS: Journal of Common Market Studies*, *60*(6), 1611–1628. 10.1111/jcms.13335

[CIT0069] Woollard, C., et al. (2022). *EU migration and asylum funds for third countries*. European Parliament. https://www.europarl.europa.eu/thinktank/en/document/IPOL_STU(2022)737870

[CIT0070] Yassen, A. O., & McGee, T. (2024). “We Fund Their Political Projects”: Refugee and IDP Rentierism in the Kurdistan Region of Iraq. *POMEPS Studies*, *50,*97-105.

[CIT0071] Zardo, F. (2022). The EU trust fund for Africa: Geopolitical space making through migration policy instruments. *Geopolitics*, *27*(2), 584–603. 10.1080/14650045.2020.1815712

[CIT0072] Zolberg, A. R. (1997). Global movements, global walls: Responses to migration, 1885–1925. In *Global history and migrations*. Routledge.

